# Conducting Web-Based Experiments for Numerical Cognition Research

**DOI:** 10.5334/joc.85

**Published:** 2019-09-19

**Authors:** Arnold R. Kochari

**Affiliations:** 1 Institute for Logic, Language and Computation, University of Amsterdam, NL; 2 Donders Institute for Brain, Cognition and Behaviour, Radboud University Nijmegen, NL

**Keywords:** Numerical cognition, Response speed, Response accuracy

## Abstract

It is becoming increasingly popular and straightforward to collect data in cognitive psychology through web-based studies. In this paper, I review issues around web-based data collection for the purpose of numerical cognition research. Provided that the desired type of data can be collected through a web-browser, such online studies offer numerous advantages over traditional forms of physical lab-based data collection, such as gathering data from larger sample sizes in shorter time-windows and easier access to non-local populations. I then present results of two replication studies that employ classical paradigms in numerical cognition research: the number-size congruity paradigm and comparison to a given standard, which also included a masked priming manipulation. In both replications, reaction times and error rates were comparable to original, physical lab-based studies. Consistent with the results of original studies, a distance effect, a congruity effect, and a priming effect were observed. Data collected online thus offers a level of reliability comparable to data collected in a physical lab when it comes to questions in numerical cognition.

## Introduction

Web-based data collection, whereby participants take part in a research study remotely from their own computer, has gained prominence in psychology in the past decade. This is due to its clear advantages: easy access to larger and more diverse samples and speed of data collection (see e.g., Gosling & Mason, 2015; Stewart, Chandler, & Paolacci, 2017; Woods, Velasco, Levitan, Wan, & Spence, 2015, for reviews). A number of tools have been developed to facilitate the building of experiments with careful timing of experimental stimuli display and accurate response recording within web-browsers. This has made web-based data collection suitable for many experimental paradigms typical in cognitive psychology. Likewise, there are now several services offering participant-recruitment from broad participant pools. A number of classical effects in cognitive psychology have been successfully replicated with data collected from participants’ web-browsers (e.g., Crump, McDonnell, & Gureckis, 2013; Semmelmann & Weigelt, 2017; Zwaan et al., 2018), validating them as viable tools for hypothesis testing. One research area in which this type of data collection can be useful is numerical cognition research, a subfield within cognitive psychology^1^ which will be the focus of the present review. Being able to reach a culturally diverse set of participants (e.g., with different traditions of teaching children to count) and to collect data easily in small variations of the same set-up (e.g., multiplication problems with differing levels of difficulty) are advantages that are especially useful in answering questions in numerical cognition.

Technological advancements have allowed for the collection of increasingly sophisticated types of data through participants’ web browsers. For a long time web-based studies collected data in the form of questionnaires and survey answers (Birnbaum, 2000; Gosling, Vazire, Srivastava, & John, 2004). Collecting reaction time (RT) data, whereby participants use their own keyboard keys to react to visually or auditorily presented stimuli, has become easier and more prominent as modern browsers (and computers) have become capable of presenting such stimuli with a reasonable timing control (de Leeuw & Motz, 2016; Hilbig, 2016; Semmelmann & Weigelt, 2017). Moreover, with the proliferation of flexible data-collection tools that require less programming knowledge (such as those which are reviewed below), expensive, custom-based software or vast programming skills are no longer necessary for data collection. The most recent developments also allow for tracking the trajectories of participants’ mouse movements (Mathur & Reichling, 2018) and recording audio and video (Semmelmann, Hönekopp, & Weigelt, 2017; Semmelmann & Weigelt, 2018). Web-based data collection methods have started being used within numerical cognition research as well; for example, studies looked at numerical information recall performance (Eriksson & Lindskog, 2017), number-line estimation (Landy, Silbert, & Goldin, 2013), mathematical anxiety scores (Cipora, Willmes, Szwarc, & Nuerk, 2018; Ferguson, Maloney, Fugelsang, & Risko, 2015), reaction time and accuracy in visual stimulus presentation (Cipora, Soltanlou, Reips, & Nuerk, 2019; Gökaydin, Brugger, & Loetscher, 2018; Huber, Nuerk, Reips, & Soltanlou, 2017). Nonetheless, usage of these methods remains rare within numerical cognition.^2^

This review focuses on experiments aimed at reaction-time data collection conducted with adult participants (see below, *Collecting good quality data in web-based experiments*, for a short discussion of collecting data with children). The technological requirements for running such experiments are: presentation of instructions and survey questions regarding demographic data, presentation of multiple trials with precise timing, storage of information about accuracy (for a potential analysis of error rates in each condition, as is typical in RT experiments) and timing of button presses by participants. From the point of view of participant commitment, participants should be able to keep their attention on the task, understand and follow instructions, and complete the task as intended (which is something we need to be able to detect). Here, I discuss these issues with a focus on the requirements for posing typical research questions in numerical cognition.

After a short discussion of the advantages and potential problematic aspects of web-based behavioral data collection, I review the available tools for experiment building and participant recruitment. This up-to-date overview will be useful to anyone starting out with web-based data collection, as it aims to answer (or point to where to find answers to) the practical questions surrounding this topic. Thereafter, I present replications of two classical paradigms in numerical cognition research that aim to investigate whether reaction times collected in a web-based study are sensitive enough for typical experimental manipulations in this area. Experiment 1 replicates the size congruity paradigm with numerical size judgment (Besner & Coltheart, 1979; Henik & Tzelgov, 1982). Experiment 2 replicates numerical distance and priming effects in a task where participants were asked to compare a digit to a given standard (Van Opstal, Gevers, De Moor, & Verguts, 2008). Anticipating the results, in both experiments I successfully replicate the original findings. Note that an earlier web-based study by an independent group successfully replicated a number of effects in a two-digit comparison task (Huber et al., 2017); in yet another web-based study conducted simultaneously with the current project, one more classical effect in numerical cognition – SNARC (Spatial-Numerical Association of Response Codes) effect – was successfully replicated (Cipora et al., 2019). Both current and those replications demonstrate the potential utility of web-based data collection as a tool for research in numerical cognition. In the final section of this manuscript, I offer some advice on how to ensure better data quality in web-based data collection.

### Advantages of web-based data collection

One of the advantages of web-based experiments for psychological research is the speed of data collection. Once there is no restriction on the geographical location of the lab, many more participants will usually fit the inclusion criteria of a study. Thus, more people will be available to participate. There is also no restriction in terms of simultaneously available lab space or computers: each participant completes the study in their own home, and, provided there is no technical limitation from the web server, many participants can complete the same study simultaneously. Lastly, if the researcher makes use of the participant recruitment tools (described below), time is also saved on appointment management: there is no need to schedule each participant and there are no delays related to absentee participants. Speed of data collection not only saves time but also allows for data collection with samples that would not be feasible if the study was not web-based. For example, questions about variability of Approximate Number System in humans as species require extremely large sample sizes (such an investigation of Approximate Number system acuity with 10.000 participants was conducted by Halberda, Ly, Wilmer, Naiman, & Germine, 2012).

Another advantage to this geographical flexibility is that one can reach a population that is not otherwise available or accessible. Web-based data collection makes it easier to recruit non-student samples, non-WEIRD samples (Western, Educated, Industrialized, Rich, Democratic samples; Henrich, Heine, & Norenzayan, 2010), or participants with various linguistic or cultural backgrounds. With regards to the former, we should keep in mind that some level of computer literacy and access to high-speed internet and is a necessary prerequisite for participating in web-based studies, so it does not completely solve the issue with WEIRD participants (see e.g., Paolacci & Chandler, 2014; Peer, Brandimarte, Samat, & Acquisti, 2017, for demographic characteristics of online participant pools). The possibility to easily reach participants with different cultural and linguistic backgrounds facilitates the verification of cross-linguistic and cross-cultural experimental effects: one can recruit participants from different populations without conducting data collection at multiple physical locations. In numerical cognition research, such comparisons could be, for example, between cultures which have different customs for teaching children how to count (e.g., Lyle, Wylie, & Morsanyi, 2019; Miller & Stigler, 1987), or from populations which read and write from left to right as opposed to right to left, which would be relevant for a question investigating mental number line (e.g., Pitt & Casasanto, 2016; Shaki, Fischer, & Petrusic, 2009).

Yet another noteworthy advantage of running web-based experiments is the fact that experiments created for web-browsers can be more easily shared between researchers. Below, I list some of the tools for programming experiments to run in web-browsers. In most cases, the data-collection script will run on any computer with a web-browser and can be modified with any text-editing software (although it should be noted that recording the collected data will require a basic web-server or web-server simulator). Unlike many traditional experiment-building tools, there is often no need to pay for licensing. This means that a researcher can simply send the experiment files to a colleague, or upload them as part of the supplemental online materials of a study (as I do for the experiments I present in this paper). Moreover, the same data-collection script can be used both to collect data remotely from participants’ own computers as well as in a physical lab set-up. We now know that findings in psychological research in general suffer from issues of low reproducibility and replicability (Collaboration, 2015; Klein et al., 2018). Although they have not been investigated specifically within numerical cognition research, these issues are most likely present there as well. Being able to easily share data-collection scripts between different laboratories will allow for close replications of reported effects, improving robustness of findings in the field.

Finally, web-based experiments can be considerably cheaper than lab-based studies if participant recruitment services are used. Contrary to a common belief, however, this is not because the participants are underpaid (in fact, not paying participants a decent amount is an ethically questionable practice; see e.g., Fort, Adda, & Cohen, 2011; Gleibs, 2017, for a discussion of this point), but because of the costs saved on experiment administration. Web-based experiments do not require research assistants to run them, and participant-recruitment services eliminate the need to spend time on scheduling participants or administering payments to each individual participant.

### Potential problematic aspects of web-based data collection

As previously mentioned, until recently a skilled web-programmer would have been required to build a reliable web-based experiment, which was problematic. However, various free, intuitive tools built specifically for this purpose are currently available. I give an overview of these tools in the next section.

Whilst participants’ environments in traditional lab-experiments are tightly controlled, we have no oversight of participants’ environments in web-based experiments. This means that the participants may not be paying as much attention to the task at hand as we may wish (see Chandler, Mueller, & Paolacci, 2014; Necka, Cacioppo, Norman, & Cacioppo, 2016, who found that online participants are often multitasking when participating in studies). Researchers normally explicitly ask participants to be in a quiet room and to pay attention only to the task at hand. However, we have no way to enforce or check for compliance with these instructions. For research questions that claim to investigate everyday brain-function, this may actually prove to be a more realistic experiment environment – for example, when participants are asked to give approximate numerical judgments (as, for example, in Landy et al., 2013). On the other hand, for research questions investigating small effect sizes, an environment filled with distractions may result in noise that conceals the effect. Another possibility is that participants cheat – for example, in a multiplication task without a time restriction they may be solving the given task on a calculator. One can explicitly ask participants if they cheated at the end of the experiment, but we again have no way to know with certainty that they were honest. In the section *Collecting good quality data in web-based experiments* below, I give some tips on how to maximize participants’ attention during the experiment and how to filter out those that did not complete the study honestly. However, since it is impossible to completely avoid these issues, their existence must be taken into account during experiment design and interpretation stages.

The more worrying aspect of collecting reaction time data in web-browsers is the accuracy of the stimulus presentation times and of the recorded reaction times. The difficulty with timing of the presentation of visual stimuli is due to varying monitor refresh rates: in order to time a stimulus exactly, one has to specify it in such a way that it takes an exact number of refresh rates (see Elze & Tanner, 2012; Woods et al., 2015, for more detailed discussions). While in a lab set-up one can set the timing based on the known exact refresh rate of the monitor used to run the experiment, it is not possible to do so for web-based experiments as pages loaded to a web-browser do not have access to information about the refresh rate of a remote monitor. If a visual stimulus is supposed to appear or disappear at a time that does not coincide with a refresh, it will only do so during the next refresh. In case of auditory stimuli, there will also be a different delay for the different computers and speakers that participants use. However, these timing issues are not as problematic as it may seem, since the delays remain more or less stable within each experimental session, so it will be approximately the same for each trial done by a participant. Thus, it should not be problematic for within-participant designs. This is supported by the results of a study by Reimers and Stewart (2015) who tested stimulus display durations across multiple computers and browsers. They found that stimuli were often presented for around 10–20 ms longer than intended, but within-system variability was small. However, note that experimental designs that go beyond simple visual or auditory stimulus presentation might have unacceptable timing issues; for example, timing lags were found to vary substantially for different browsers and computers when synchronization of auditory and visual stimulus onset was required (Reimers & Stewart, 2016); this issue would, for example, hinder web-based administration of paradigms requiring cross-modal numerical stimulus presentation (as in Lin & Göbel, 2019).

Another problematic aspect is related to delays in reaction time recording: different keyboards will have different delays between the pressing of a key and detection of the press (Neath, Earle, Hallett, & Surprenant, 2011; Plant & Turner, 2009). There will also be delays in RT recording related to inaccuracies in web-browsers and to the processing speed of the computer. Multiple studies have compared recorded reaction times in a lab set-up and a web-browser-based collection, and they all consistently find delayed RTs for the latter of 25–100 ms (de Leeuw & Motz, 2016; Hilbig, 2016; Reimers & Stewart, 2015; Semmelmann & Weigelt, 2017). Importantly, again, the within-participant variability was stable, and therefore the delayed RTs did not affect the size of the observed differences between conditions in within-participant designs (de Leeuw & Motz, 2016; Hilbig, 2016; Reimers & Stewart, 2015; Semmelmann & Weigelt, 2017). When it comes to between-participant designs, the different delays for different participants can potentially be compensated for by testing a larger number of participants in each group (Reimers & Stewart, 2015).

One general issue with using online recruitment services is that participants are likely to complete many studies over time and, therefore, there is a high likelihood that they have experience with similar experimental paradigms or with completing artificial tasks in general. In other words, some of these participants might not be considered naive to the task (Chandler et al., 2014; Peer et al., 2017; Stewart et al., 2015). Participant naivety to the experimental manipulation is often desirable as it is an important assumption of some paradigms (see Chandler, Paolacci, Peer, Mueller, & Ratliff, 2015; Weber & Cook, 1972, for reviews of cases where participant non-naivety can lead to different effect sizes). However, typically, the effects that we are interested in in cognitive psychology, including in numerical cognition research, are robust to participant non-naivety (see Zwaan et al., 2018, for successful replications of classical cognitive psychology effects with non-naive participants). This aspect of web-based data collection is thus less problematic for numerical cognition research than it is for some other research areas.

A number of studies have successfully replicated classical effects in cognitive psychology in web-based studies: Stroop, Flanker, Simon, visual search, attentional blink, serial position, masked priming, associative priming, repetition priming, lexical decision task etc. (Barnhoorn, Haasnoot, Bocanegra, & van Steenbergen, 2015; Crump et al., 2013; Hilbig, 2016; Semmelmann & Weigelt, 2017; Zwaan et al., 2018). As already mentioned, empirical data presented in the current manuscript as well as successful replications of other classical numerical cognition effects (Cipora et al., 2019; Huber et al., 2017) extend their suitability to paradigms typical in numerical cognition research. However, as this section discusses, one should keep in mind that there are certain limitations with web-based data collection: not every lab paradigm will work well running within a web-browser or be suitable for completion in an environment with possible distractions. Experiment 2 below replicates one such paradigm that is problematic for web-based data collection – masked priming – for which browser timing inaccuracies for short latencies seem to hinder replication of the effect observed in the lab (Crump et al., 2013, experiment 7). The issue with the environment in which the study is completed remains even in case the web-browser is able to execute the experiment flawlessly.

## How to set-up a web-based behavioral experiment

Recently, a number of free community-run and fee-based commercial tools for experiment building have become available, making the creation of web-browser based experiments possible with minimal to no web-programming skills at all. Another crucial component in web-based data collection is participant recruitment, which has also became more straightforward with the launch of multiple services specifically aimed to meet this particular need. This section of the paper is intended to be an up-to-date, high-level primer regarding all practical aspects of web-based reaction time data collection. More detailed tutorials are available in the published articles and manuals for each specific tool that I refer to below.

### Building behavioral experiments for web-browsers

Table 1 provides an overview of some of the tools available for building experiments to be run in a web-browser.^3^ These tools differ in the amount of programming knowledge required, in their pre-programmed functionality, and in whether they are free. They make the task as easy as building experiments to be run in traditional physical lab-spaces (such as PsychoPy, DMDX, E-Prime, Presentation etc.). While other technologies such as Adobe Flash were used in the past, presently JavaScript in combination with HTML5 is the preferred technology as the two are supported by all modern browsers. All of the listed tools support manual scripting using basic JavaScript and HTML code (hence, some programming experience would be required), while some also offer a graphical user interface (hence, no programming experience is needed). Because participant recruitment is done separately from experiment building (although the commercial tools also offer help with participant recruitment), it does not matter which exact tool is used for experiment creation.

**Table 1:** Overview of some of the available tools for building cognitive psychology experiments to run in web-browsers. This list is not comprehensive, as often development is discontinued and new tools frequently appear.

Name	Website	Free	Graphical interface	Introduction paper

jsPsych	jspsych.org	yes	no	de Leeuw (2015)
lab.js	lab.js.org	yes	yes	Henninger, Shevchenko, Mertens, Kieslich, and Hilbig (2019)
PsychoPy/PsychoJS	github.com/psychopy/psychojs	yes	yes	Peirce et al. (2019)
PsyToolkit	psytoolkit.org	yes	no	Stoet (2017)
Gorilla	gorilla.sc	no	yes	Anwyl-Irvine, Massonnié, Flitton, Kirkham, and Evershed (2019)
LabVanced	labvanced.com	no	yes	–

For the experiments presented in this paper, I used jsPsych (de Leeuw, 2015); I will briefly describe what experiment building is like with this tool, by way of example. jsPsych is a free, community-built and maintained JavaScript library (i.e., a collection of pre-written functions that can be used by themselves or in addition to other code written in JavaScript) that is optimized for accurate stimulus display and reaction-time data collection. Due to its transparent modular architecture, jsPsych is suitable as an experiment creation tool even for researchers with little to no programming experience. Each experiment script is an HTML page with JavaScript code; it is edited with a text editor and run by opening the same HTML file with a web-browser. Within this JavaScript code, an experimenter defines each display (page) of the experiment, the stimuli within these displays, the timing, what the participants are allowed to do (e.g. that they proceed to the next display by pressing only a certain key or that the next display is automatically shown after a certain time), and the order of presentation (or randomization parameters). jsPsych takes care of rendering each of the displays according to the set parameters, recording the answer, and sending the collected data to the determined place for storage (see below for more on this). In the most recent release of jsPsych (at the time of writing this paper), text, images, audio and video can be handled as stimuli by pre-programmed functions in jsPsych; multiple types of survey-question responses, button presses, reaction times of button presses and mouse clicks can be collected. For those new to jsPsych, detailed tutorials and a basic experiment template that can be used as a starting point is available in the supporting documentation for jsPsych (with additional experiment scripts that can be used as templates shared by the community).

### Hosting the experiment and storing collected data

Once we figure out the stimulus display and data recording script, the next step is to place it on a web server where participants can access it (i.e., it needs to be hosted somewhere) and arrange for the storage of recorded data. What we would like in the end is a link which participants can follow to take part in the experiment; this link is then given to the recruited participants, for example, through participant recruitment tools. There are multiple options for hosting and data storage. Each of the experiment building tools listed in Table 1 has a help page with detailed suggestions for how to arrange data storage. One way is to host an experiment on a rented or university web hosting service and store the data there; this option requires some basic knowledge of configuring web-servers. There are also independent services for experiment hosting and data storage (e.g. psiTurk – https://psiturk.org/ and JATOS – http://www.jatos.org/, both of which are free and community-run). The commercial experiment building tools that I list above offer to take care of it for you in exchange for a fee.

If you choose to host the experiment on a web server yourself, there are multiple ways to get such a server. Many universities provide personal web-hosting space for their employees that has some basic functionality, which would normally be sufficient for running web-based experiments; that is exactly what was used for the hosting and data collection of the experiments described here. Another option is to rent a hosting space from one of a large variety of companies offering web hosting. In both of these cases the researcher needs to ensure that the server is reliable and that the personal data of participants, if such data is collected, is stored securely as per local requirements. The easiest way to store data collected with jsPsych is as a separate CSV file for each participant. This method requires only that the web-server on which the experiment is hosted supports PHP, which most servers will do by default. The data can be saved, for example, throughout the experiment at the end of each experimental trial. More advanced users can configure data storage in databases such as MySQL.

If it only uses text stimuli (as was the case for the experiments presented here), an experiment built using jsPsych is loaded as a whole before it shows anything to the participant and only connects to the web-server to save the collected data. Thus, there will be no delays related to the internet connection speed of the participant. In case the experiment displays images, audio, or video files, it is also possible to make sure that it only starts after all necessary files are loaded to the computer memory to avoid any delays related to retrieving them from the web-server: jsPsych allows preloading of the media.

### Participant recruitment tools

The next step in the process is to recruit participants that will complete the experiment. One way would be to find people willing to take part in the experiment for free, recruiting them, for example, through social media. This would be the best or perhaps the only way forward for those aiming to collect data from thousands of participants, and would also require giving participants some motivation other than a financial incentive to take part (for example, Halberda et al., 2012 collected data by presenting it as a game and offering to give them a score at the end). Here, I focus on another way to recruit participants, namely through crowdsourcing platforms where they come specifically to complete tasks in exchange for a financial reward. This is most suitable for a typical study in numerical cognition, since it is not necessarily interesting enough for participants to just want to do it in their leisure time (sometimes one could think of incentives such as finding out how well one does in comparison to the general population^4^), and would require only dozens or hundreds of participants.

The crowdsourcing platform that presumably has the largest pool of participants is Amazon Mechanical Turk (https://www.mturk.com) (e.g., M. Buhrmester, Kwang, & Gosling, 2011; M. D. Buhrmester, Talaifar, & Gosling, 2018). Amazon Mechanical Turk is a marketplace where any sorts of tasks that can be completed remotely, on a computer, are given and taken up by participants. The other presently prominent platform is Prolific Academic (https://prolific.ac/) which is geared specifically towards academic research studies (Palan & Schitter, 2018; Peer et al., 2017). As this is a new industry, a number of other similar platforms appear and close down from time to time (see e.g., Peer et al., 2017; Vakharia & Lease, 2015). Both of the above-mentioned platforms allow for some filtering of eligible participants based on basic demographic data that they fill in: for example, based on age, education level, or native language. Besides the payment to the experiment participant, the researcher pays a fee to the crowdsourcing service.

The data of the experiments reported in the present paper were collected using Prolific Academic (henceforth, simply Prolific), so I will also shortly discuss how this particular service works as an example. On Prolific, the researcher creates a study specifying the participant eligibility criteria, the amount of time the experiment should take, and the amount to be paid, and provides a link that participants should follow to complete the study. A short description of the study is also given to participants, based on which they can decide whether to take part or not. Importantly, the researcher also has an opportunity to include, restrict to, or exclude participants that have taken part in previous studies they have offered. Prolific has a minimum required hourly rate to be paid to participants (£5 at the time of writing this paper), and charges a fee for each of the participants (30% at the time of writing this paper). The researcher also has the possibility to give each individual participant a bonus based on their performance in the study.

Prolific currently has just over 40,000 registered participants, all from OECD countries (people living in other countries are not allowed to register as experiment participants). The participants only have the opportunity to complete a study if they are eligible for it based on their demographic information. If participants choose to take part in a study, they follow the link that is given; Prolific logs the time of the start of the experiment. The experiment is run either in a window with a Prolific heading at the top or in a new window. As a way to confirm that the participant has indeed completed the study, the researcher puts a study-specific link (generated by Prolific) on the last page of the experiment. This link takes the participant back to Prolific, which logs the time of completion. The experimenter has to approve the submission (i.e., verify that the participant undertook the study honestly) before the participant gets paid. After the study is completed, demographic data for participants that took part in it, along with the start and end time for each participant, can be downloaded from Prolific.

## Replications of classical behavioral experiments in numerical cognition

In this section, I present replications of two classical and widely used paradigms in numerical cognition research. Observing the comparable effects in a web-based study and one conducted in a traditional lab-based set-up would support the viability of web-based data collection tools for testing hypotheses in numerical cognition.

Experiment 1 was conducted as part of a different research project and the results are primarily reported in another paper (Kochari & Schriefers, in preparation). Here, I only briefly describe it for the purpose of demonstrating the feasibility of getting sufficient quality reaction time data in a web-based experiment. For the same reason, this is not a direct replication of any particular study, but rather a replication of the effects in general. Experiment 2 is conducted as a direct replication of part of a study by van Opstal et al (Van Opstal et al., 2008). Besides the presentation of stimuli and the recording of button-press reaction times, this experiment also includes a subliminal priming manipulation. These replications are only meant as demonstrations of technical possibilities, so I do not offer an interpretation of the effects themselves or their theoretical implications. A successful replication would, however, also demonstrate that these effects, whatever they mean, are robust, since they can be observed in a less controlled environment than traditional physical labs.

The scripts used for data collection, commented data analysis scripts, and all data that were collected are available for inspection and download at https://osf.io/dy8kf/. Note that these scripts can be easily modified for collecting data in similar studies.^5^

### Experiment 1: Size congruity effect

In Experiment 1, I replicate a size congruity effect that was first reported several decades ago (Besner & Coltheart, 1979; Henik & Tzelgov, 1982). Since then, the size congruity paradigm in its original and modified forms has been used to answer numerous questions about number and magnitude perception. In this paradigm, participants are presented with two numbers (e.g., digits, number words etc.) on two sides of the screen and are asked to press a button corresponding to the side of the screen with the numerically larger digit. However, the two numbers that are presented can be of a different physical (font-) size: the numerically larger digit can be physically larger (congruent condition), the numerically larger digit can be physically smaller (incongruent condition), or they can be of equal physical size (neutral condition). Robust congruity effects are typically observed: people are faster at giving responses and make fewer mistakes in the congruent in comparison to the incongruent condition.

Another variable that is traditionally manipulated in this paradigm is how big the difference between two stimuli is: the numerical difference between the two presented numbers can be large or small (e.g., 2–4 vs. 2–8; I refer to this factor as *numerical distance*) or the physical (font-) size difference between the two presented numbers can be large or small (I refer to this factor as *size distance*). Distance is a relevant factor here since we know that it is more difficult to distinguish values that are closer to each other (e.g., 2–4) than values that are further away from each other (e.g., 2–8) (Moyer & Landauer, 1967). In the size congruity paradigm, numerical and size distance have been found to modulate the congruity effect (see e.g., Cohen Kadosh, Henik, & Rubinsten, 2008; Henik & Tzelgov, 1982; Kaufmann et al., 2005; Pinel, Piazza, Le Bihan, & Dehaene, 2004; Tzelgov, Meyer, & Henik, 1992). The congruity and distance effects in this paradigm have been interpreted as indicating the automaticity of magnitude processing, since information about the irrelevant dimension modulates performance in the relevant dimension, as well as being used as an argument for the existence of some shared magnitude-processing mechanism (e.g., Cohen Kadosh & Henik, 2006; Cohen Kadosh, Lammertyn, & Izard, 2008; Santens & Verguts, 2011; Tzelgov et al., 1992). As mentioned above, it is not my aim here to address the issue of interpreting the effects themselves. Instead, I focus on whether the basic effect is replicable in a web-based set-up.

In the present experiment, participants judged the numerical value of the presented Arabic digits. I manipulated congruity, numerical distance, and size distance. Based on the results of the classical experiments reporting the size congruity effect (Henik & Tzelgov 1982), I expected to obtain a main effect of congruity, a main effect of numerical distance (because overall, numbers that are further apart from each other should be easier to judge), as well as an interaction between congruity and physical size distance (because disruption of judgment in the incongruent condition will be stronger when the difference in the irrelevant physical (font-) size dimension is larger).

#### Method

##### Participants

Given that previous studies were able to detect the size congruity effect with 10–20 participants (e.g., Cohen Kadosh, Henik, & Rubinsten, 2008; Henik & Tzelgov, 1982; Kaufmann et al., 2005), I aimed for a sample size of around 25 participants in this task. Participants were recruited via Prolific.ac. The following inclusion criteria were applied: age 18–25, speaking English as a native language, being born and currently living in the UK. Participants received £1.30 for participation. Participants were excluded from the analyses if they spent less than 10 seconds reading the task instructions or if they gave incorrect responses in more than 15% of trials.

Twenty-six participants completed the study in full. Two participants were excluded because they gave incorrect responses in more than 15% of trials; one further participant was excluded due to reading the instructions for less than 10 seconds. Thus, 23 participants in total were included in the analyses (12 female, 11 male; 3 left-handed; 7 students; average age: 27 [range 19–34]; average time spent on the task: 6:03 minutes [range 4:56–10:47]).

##### Stimuli

Eight digit pairs were included: four pairs had a numerical distance of 2 units (*2-4, 3-5, 5-7, 6-8*) and four pairs had a numerical distance of 4 units (*2-6, 3-7, 4-8, 5-9*). Each digit pair was displayed in congruent (the digit in the larger font size is numerically larger) and incongruent (the digit in the larger font size is numerically smaller) conditions. Each digit pair was also displayed in two levels of physical size distance: the font sizes were either 64 pt and 72 pt (*small size distance*) or 55 pt and 72 pt (*large size distance*). Finally, each of the trials was repeated twice, once with the larger number on the left side of the screen and once with the larger number on the right side of the screen. This resulted in 8 (digit pairs) * 2 (congruity levels) * 2 (physical size distance levels) * 2 (sides of the screen) = 64 trials in total. I addition, I included 16 neutral trials (both digits were displayed in font size 64 pt) and 16 filler empty trials, in which participants saw a fixation cross, as in regular trials, but in this case it was followed by a blank screen for 1850 ms. In total participants saw 96 trials. While the neutral condition was present in this experiment, I did not include it in the statistical tests, as assessing whether congruity is driven by facilitation or interference (as in e.g., Cohen Kadosh, Henik, & Rubinsten, 2008) was not my goal.

##### Procedure

The experiment was implemented using jsPsych (de Leeuw, 2015). Prior to the experiment, participants agreed to data collection and filled in a questionnaire asking for basic demographic information. Throughout the experiment, they advanced using the space key or the experiment advanced automatically between experimental trials. Participants were instructed to indicate whether the number on the left or on the right was numerically larger by pressing buttons “Q” or “P” correspondingly. They were asked to do so as quickly as possible. An example was given, and they had a chance to practice making the judgments in 4 practice trials.

Each trial started with a fixation cross (‘+’) displayed for 150 ms in the middle of the screen. It was followed by a display on the screen where one digit was displayed to the left and another digit to the right of the center. The digits were displayed in Arial font. The digits remained on the screen until the participant gave a response or, if no response was given, for 1850 ms. In case of no response, the experiment automatically advanced to the next trial. The inter-trial interval was a random number between 700 and 1200 ms.

The experiment was divided into 2 blocks of 48 trials, and the participants had a chance to rest between the blocks. The order of trials was fully randomized. The data for each participant was saved as a separate .csv file on the web-server where the experiment was hosted; this file was updated after each trial.

#### Results

Participants gave incorrect responses in in 3.7% of trials in total (including trials where no response at all was given). This error rate is within the normal error range for this paradigm (approximately 1–6% based on the studies reviewed above). The general RT level was approximately within the 500–650 ms range, which also falls with the normal range of RTs for this paradigm (e.g., it is somewhat faster than the RTs observed by Henik & Tzelgov, 1982, but somewhat slower than those observed by Cohen Kadosh, Henik, & Rubinsten, 2008).

**Figure 1:**
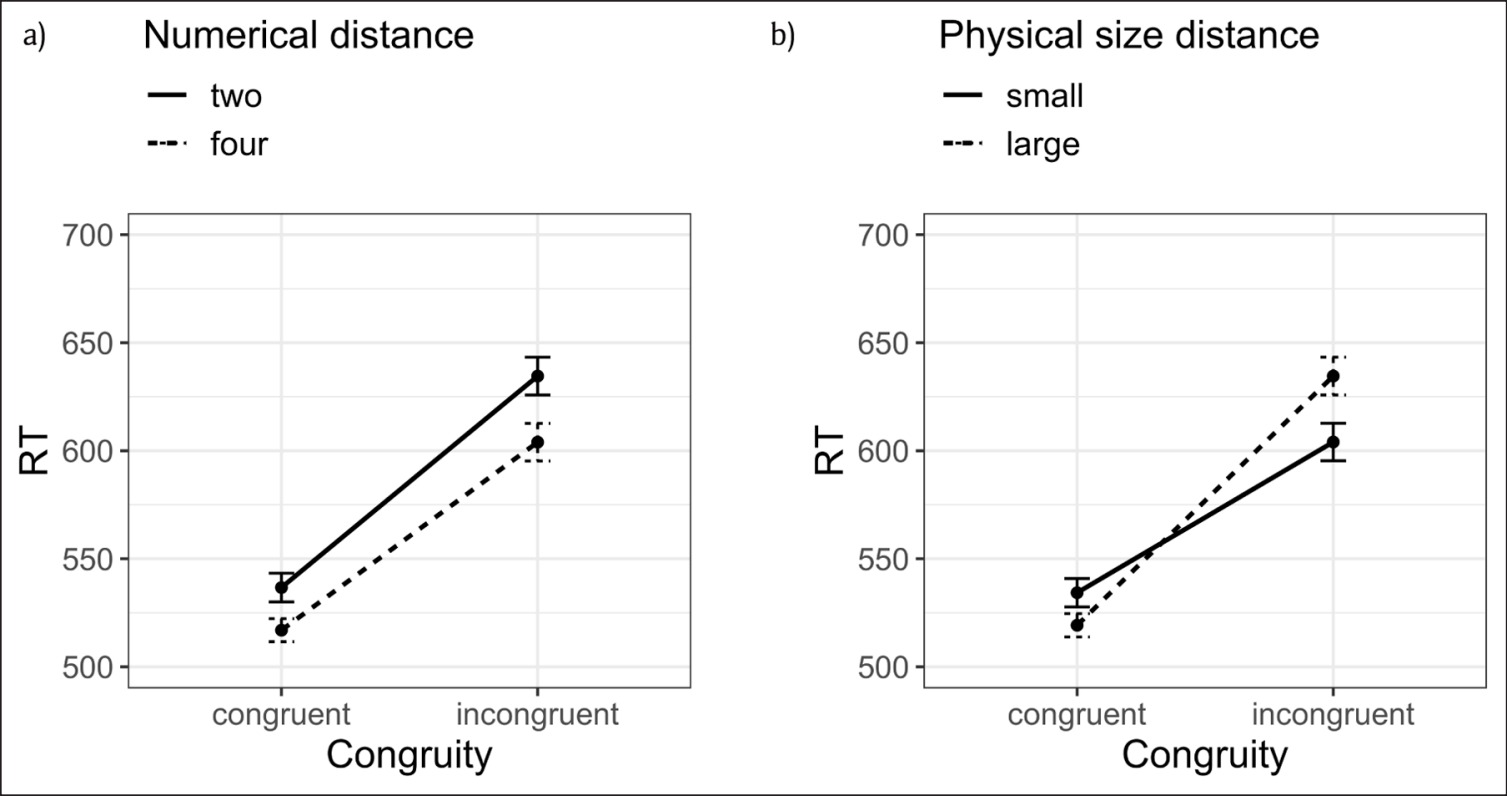
Mean RTs per congruity in Experiment 1. The error bars depict the standard error value. **(a)** Split by numerical distance, collapsing different physical size distances. **(b)** Split by physical size distance, collapsing different numerical distances.

Only RTs of correctly answered trials were included in the analyses. Prior to the analyses, I excluded all trials in which the reaction time was too short (<250 ms) to have been initiated after processing the target, as well as reaction times shorter or longer than 2 standard deviations from the mean for a given participant for a given condition.^6^ This resulted in the exclusion of 8.2% of trials. The resulting RTs, split by congruity and numerical distance, are shown in Figure 1a. The same RTs, but this time split by congruity and physical size distance, are shown in Figure 1b.^7^

I performed a 2 (congruity: congruent or incongruent) × 2 (numerical distance: two or four units) × 2 (physical size distance: small or large) within-subjects ANOVA on mean correct RTs. All the predictions were borne out by the data. Participants gave faster responses to congruent trials (526 ms) in comparison to incongruent trials (612 ms, difference 86 ms) [*F*(1,22) = 60.7, *p* < 0.0001, \eta _p^2 = 0.73]. This effect size is comparable to that observed for the congruity effect in Henik and Tzelgov (difference 116 ms; \eta _p^2 = 0.9; Henik & Tzelgov 1982^8^). I also observed a significant main effect of numerical distance [*F*(1,22) = 8.83, *p* = 0.007, \eta _p^2 = 0.28] which was somewhat smaller than the one observed in Henik and Tzelgov (\eta _p^2 = 0.68). Finally, I observed a significant interaction between physical size distance and congruity [*F*(1,22) = 15.24, *p* = 0.0007, \eta _p^2 = 0.40; this factor was not manipulated in the study by Henik and Tzelgov]. None of the other effects were significant.

### Experiment 2: Numerical distance and priming effects in comparison to standard

In Experiment 2, participants compared a digit to a pre-determined standard (in the present case, the standard was the number 5) and pressed one button in case it was greater than the standard and another button in case it was smaller than the standard. Here too, reaction times and error rates were measured. The theoretical motivation of this paradigm was similar to those that use the size-congruity paradigm (Experiment 1): it is known that the further away the two digits to be compared are, the shorter the reaction times get and the fewer errors participants make; hence, a distance effect should be observed (e.g., Dehaene, Dupoux, & Mehler, 1990; Hinrichs, Yurko, & Hu, 1981; Sekuler, Rubin, & Armstrong, 1971). In addition, in this experiment there was a masked prime manipulation – another digit was presented prior to the target digit that participants judged (Dehaene et al., 1998).

Specifically, in this experiment I replicate a study by van Opstal and colleagues (Van Opstal et al., 2008) that looked at several different effects: the effect of numerical distance between the target digit and the standard digit to which it was to be compared (following their terminology, I will refer to this effect as *comparison distance effect*), the effect of numerical distance between the target digit and the prime digit (following their terminology, I will refer to this effect as *priming distance effect*), and, finally, the *congruity-priming* effect, which refers to the difference between the trials where both the prime and target digit would result in the same response (e.g. both are above standard or both are below standard) and the trials where the prime and the target digit would result in a different response (e.g. the prime is below the standard whereas the target is above the standard). The reasoning of the original study was that unlike the comparison distance effect, which can be explained either by the placement of numbers on an analogue continuum or by response-related processes, the priming effect excludes the response-related processes explanation (see Van Opstal et al., 2008, for the goals of the study). While the original study also used the same paradigm to look at the effects with letters of the alphabet, here I will only replicate their experiment with digits. Van Opstal and colleagues performed the same experiment twice (Experiment 1 and Experiment 2), obtaining the same result; when drawing comparisons to their results below, I will provide the resulting RTs in both of their experiments.

Masked (also referred to as *subliminal*) priming studies require the precise timing of the stimulus display: the prime is usually displayed for some short amount of time, and we need to be sure that the prime has indeed appeared on the monitor and for the specifically defined amount of time which in itself might be an experimental variable. This is challenging in a web-based set-up where we have no control over the exact apparatus that participants are using, and therefore cannot synchronize with their monitor refresh rates. One web-based masked priming study by Crump et al (Crump et al., 2013, Exp. 7) attempted to replicate an effect of the compatibility of prime arrows with target arrows (e.g., ‘»’ primed by ‘»’ or ‘«’) in a task where participants simply press a button corresponding to the direction of the arrow. They manipulated the duration of the prime (in 6 steps from 16 to 96 ms) as an experimental factor, expecting the shortest prime durations to result in a negative priming effect (longer RTs after compatible primes) and the longest prime durations to result in a positive priming effect (shorter RTs after compatible primes). They only successfully replicated the priming effects expected for the two longest prime durations, but not the priming effects expected for the shorter prime durations, which were also all trending in the positive direction instead of the expected negative. This was likely due to the fact that with prime durations as short as 16 ms, due to not being synchronized with monitor refresh rates, the primes were sometimes displayed for too long. However, another replication of this effect, which used a different JavaScript library to administer the experiment, did observe the expected positive and negative priming effects (Barnhoorn et al., 2015, Exp. 3).^9^ Nonetheless, in case the exact duration of the prime display is important for the research question at hand, web-based data collection is not an advised tool since we cannot control it well enough. Web-based data collection would be suitable if it were acceptable for the prime to be displayed for +/–1 or 2 frames per second longer (which for an average monitor means +/–16 or 32 ms). In the present experiment, the exact duration of the prime was not an experimental factor for the study at hand; moreover, the duration of the masked prime in the study of van Opstal et al was 83 ms – the duration for which both Crump et al and Barnhoorn et al successfully observed priming.

Since this is a direct replication, the present experimental procedure was the same as that described in the van Opstal et al study number task (Van Opstal et al., 2008). Whenever I diverged from it, I explicitly mention what exactly was done differently.

#### Method

##### Participants

Participants were recruited via Prolific, with the same inclusion and exclusion criteria as for Experiment 1, except for one additional inclusion criterion. Namely, in addition participants were not allowed to have completed more than 50 other studies on Prolific. This was done to facilitate participant naivety, which has been raised as a potential issue with participant recruitment through online crowdsourcing services (Chandler et al., 2014; Stewart et al., 2015, see the section *Collecting good quality data in web-based experiments* for a more detailed discussion).

Eighty-one participants completed the experiment across two response button mappings (see below for explanation). They received £ 2.50 for participation. Seven participants were excluded from the analyses due to having given incorrect responses in more than 15% of trials, and two further participants were excluded due to reading the instructions for less than 10 seconds. This resulted in 72 participants being included in the analyses presented below (41 female, 31 male; 13 left-handed; 34 students; average age: 26 [range 18–35]; average time spent on the task: 15:24 minutes [range 11:41–28:22]).

The number of participants for the present study was determined in such a way as to be comparable to that of van Opstal et al. This study included fewer trials than the original study because it is more difficult to ensure participants attention for longer periods of time in a web-based study (see below, *Collecting good quality data in web-based experiments*, for a discussion). Because there were fewer observations per experimental condition per participant here, I increased the total number of participants in such a way as to end up with approximately the same number of observations per experimental design cell as van Opstal et al.

##### Stimuli

The stimuli in this experiment were same as in the original van Opstal et al study number task. That is, all numbers from 1 to 9, except 5, functioned as both primes and targets, resulting in 64 different prime-target combinations. However, here the participants saw fewer repetitions of each of the combinations: whereas participants in van Opstal et al saw each prime-target combination 10 times (resulting in 640 experimental trials in total), in the present study participants saw only 4 repetitions of each combination (resulting in 256 experimental trials in total).

##### Procedure

The data were again collected using jsPsych (de Leeuw, 2015). Prior to the experiment, participants agreed to data collection and filled in a questionnaire asking for basic demographic information. Participants were instructed to indicate as quickly as possible whether a number that they would see following ‘###’ was higher or lower than 5. There were two versions of the experiment with different response-button mappings: 38 of the participants included in the analysis were instructed to press ‘Q’ if the number was lower than 5 and ‘P’ if the number was higher than 5; 34 of the included participants received the reverse instructions. The presence of prime digits was not mentioned in the instructions. After reading the instructions, participants completed 8 trials as a practice. In these trials, they received feedback about the correctness of the given response immediately after they gave the response. No feedback was provided during the actual experiment.

The stimuli were displayed in the middle of the screen, in white Courier 36 pt font on a black background (Van Opstal and colleagues presented stimuli in font size 32 pt). Each trial started with a fixation cross (‘+’) displayed for 500 ms. This was followed by a mask (‘###’) displayed for 100 ms, a prime digit displayed for 83 ms (this would correspond to 5 frames on a monitor with a refresh rate of 60 Hz), and another mask displayed for 100 ms. Finally, the target digit itself was presented until the participant gave a response or for a maximum of 2000 ms. If no response was given, the experiment automatically advanced to the next trial (van Opstal et al did not restrict the time participants had to give a response; I diverged from this in order to make it impossible for the participants to switch their attention to something else during the experiment). The inter-trial interval was 1000 ms.

The experiment was divided into 4 blocks, with the possibility for participants to rest between blocks. In each block, participants saw each of the prime-target combinations once.

#### Results

Participants gave 3.26% incorrect responses on average (including trials where no response at all was given; van Opstal et al had an error rate of 6.9% in Experiment 1 and 6.5% in Experiment 2). Overall, the reaction times in the present experiment were ≈90–120 ms longer than in the van Opstal et al data. This is likely due to the fact that the participants in their study completed significantly more trials than in the present study (1280 [640 for number task and 640 for letter task] vs. 256) which meant they were better trained in the task.

Only the reaction times of correctly answered trials were included in the analysis. Before analysing reaction times, responses that were too fast (<250 ms) to have been initiated after having processed the target digit were excluded; this resulted in the exclusion of 0.05% of trials (van Opstal et al do not report whether they performed an RT cleaning procedure, but I do not consider these RTs meaningful; I did not exclude comparatively long reaction times since the skewed distribution of RTs is likely the reason why Van Opstal et al conducted their analyses on the median RTs). Following van Opstal et al, I also use the median RTs as the dependent variable and performed the same analyses, except that I do not have ‘task’ as an experimental factor (they had two tasks: number comparison and letter comparison).

##### Comparison distance effect

**Figure 2:**
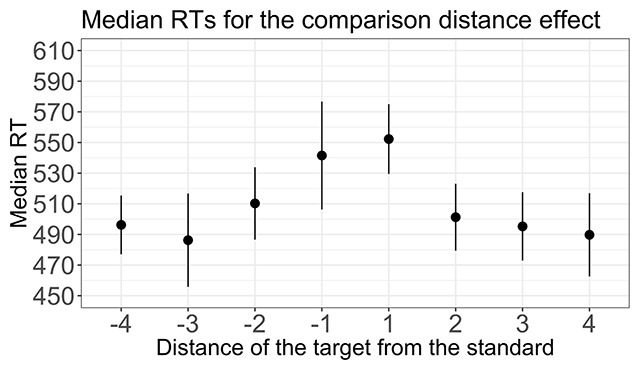
Median reaction times (RTs, in milliseconds) for the comparison distance effect. The error bars represent a 95% confidence interval.

In order to avoid a confound with the priming distance effect, only trials with identical primes and targets were included in this analysis. Figure 2 shows the comparison distance effect. As expected, RTs decreased with the increasing distance of the target digit from the standard. I performed a 2 (size: below/above the standard) × 4 (comparison distance, absolute value: 1, 2, 3, or 4) within-subjects ANOVA. Consistent with the results of van Opstal et al, I observed a significant main effect of comparison distance [*F*(3,213) = 10.65, *p* < 0.0001, \eta _p^2 = 0.13]. The size of the observed comparison distance effect was smaller than that reported by van Opstal et al. (\eta _p^2 = 0.38).^10^ Also consistent with the results of van Opstal et al, there was no main effect of size [*F*(1,71) = 2.5, *p* = 0.11, \eta _p^2 = 0.03] and no interaction between comparison distance and size [*F*(3,213) = 0.38, *p* = 0.17, \eta _p^2 = 0.005].

##### Congruity-priming effect

In this analysis, I looked at the effect of congruity of the prime and target digit – whether they would result in the same response or in a different response. Here, trials with identical primes and targets were removed to avoid confounding perceptual priming. I performed a 2 (size: below/above the standard) × 2 (congruity) within-subjects ANOVA on median RTs. There were significantly faster reaction times for the congruent (528 ms) in comparison to the incongruent (549 ms, difference 21 ms) prime-target pairs (main effect of congruity: *F*(1,71) = 58.44, *p* < 0.0001, \eta _p^2 = 0.45). This is consistent with the results of van Opstal et al (the congruity effect was 26 ms in Experiment 1 and 24 ms in Experiment 2). In addition, the congruity effect was larger for the digits above the standard (28 ms) than for the digits below the standard (17 ms) (interaction of congruity and size: (*F*(1,71) = 4.4, *p* = 0.03, \eta _p^2 = 0.05) which is also consistent with the findings of van Opstal et al (differences: 30 and 22 ms in Experiment 1, 28 and 21 ms in Experiment 2). Finally, in the present study, but not in van Opstal et al, regardless of the congruity, the reaction times were faster overall for the primes below the standard than above the standard; however, this difference was small (difference 5 ms; main effect of size: *F*(1,71) = 4.34, *p* = 0.04, \eta _p^2 = 0.05).

##### Priming distance effect

**Figure 3:**
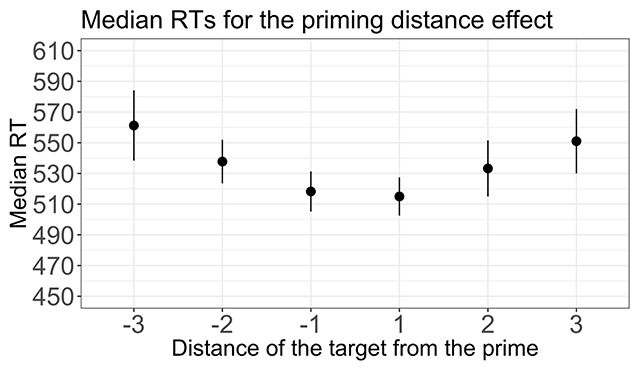
Median reaction times (RTs, in milliseconds) for the priming distance effect. The error bars represent a 95% confidence interval.

Only congruent trials were included in this analysis. Figure 3 shows the priming distance effect. Diverging from the analysis reported by van Opstal et al, I performed this analysis including targets both below and above standard, whereas van Opstal et al. only included targets above the standard (they did so for an independent reason related to the fact that they were interested in comparing priming effects for numbers and letters). I performed a 3 (priming distance, absolute value: 1, 2 or 3) × 2 (size: below/above the standard) within-subjects ANOVA on median RTs. Consistent with the results of van Opstal et al, I observed a main effect of priming distance [*F*(2,142) = 28.3, *p* < 0.0001, \eta _p^2 = 0.28]. The size of this effect is comparable to that reported by van Opstal et al (\eta _p^2 = 0.23). There was no effect of size [*F*(1,71) = 0.85, *p* = 0.3, \eta _p^2 = 0.01] and no interaction between priming distance and size [*F*(2,142) = 0.23, *p* = 0.7, \eta _p^2 = 0.003].

### Discussion of the replication results

Both of the presented replications demonstrate that the reaction time effects previously reported in traditional lab studies can be successfully observed when collecting data from participants’ web-browsers remotely, confirming numerous earlier studies in other sub-fields of cognitive psychology (Crump et al., 2013; Semmelmann & Weigelt, 2017; Zwaan et al., 2018). In Experiment 1, all expected effects in a classical variant of the size congruity paradigm were observed, whereas in Experiment 2 all expected effects in a comparison to the given standard task were observed in a direct replication of van Opstal et al (Van Opstal et al., 2008). The general error rates and reaction times were also within the range of expected values. The results of these experiments convincingly show that getting good quality data is feasible in at least relatively simple web-based numerical cognition experiments.

I draw comparisons between the present results and the results of the original studies based both on statistical significance patterns and on observed effect sizes. However, we should keep in mind that neither of these measures is a good proxy for such comparisons. The statistical significance is a binary, and therefore not very informative measure, whereas observed effect sizes are not reliable since both the present and the original studies had low sample sizes and, therefore, do not yield a good estimate of the real effect size.

In Experiment 2, a masked priming manipulation was included in addition to target digit manipulation. Although I do successfully replicate the priming effects observed by van Opstal et al, this is likely because the actual duration of the prime did not matter for the this effect. As discussed above, we do not know how long exactly the prime stimuli were displayed since, with JavaScript, there is no way of finding out exactly how long the stimulus was displayed on the screens of the participants’ computers. While in a traditional lab set-up we would be able to set the duration of the stimuli in terms of the number of refresh rates on the monitor used for testing, we cannot do so in this case. Researchers should, therefore, approach data collection for studies in which the exact duration of the prime matters carefully.

## Collecting good quality data in web-based experiments

While psychologists are already trained to interact with participants in a lab-based setting in such a way as to maximize the quality of the collected data, moving to a web-based set-up introduces a number of new challenges. In this section, I will outline some solutions to common worries associated with web-based data collection.

### Ensuring participants have suitable equipment

In order to decrease the noise in the collected data due to differences in the equipment used by participants and in order to make sure that the stimuli presentation proceeds in the intended way, we may want to exclude some devices. For example, if the experiment contains audio stimuli, one way to ensure the participants are hearing these stimuli and that they hear them at the intended volume could be to implement a password presented auditorily at the beginning of the experiment, but also repeat it later on to make sure that the equipment stays the same. The same approach can be used if the monitor needs to display certain brightness contrasts and colors (see Woods et al., 2015, for a more detailed discussion).

### Ensuring participants are doing the study honestly

This is perhaps the most worrisome aspect of web-based data collection for psychologists: participants may simply click through the experiment, respond at random, or give dishonest responses. There a number of simple checks that can be implemented in the experiment. One could use a combination of these checks that suits a study best. If participants respond at random in a straightforward task such as the comparison of numbers, it will be clear from the chance-level performance (for example, two participants in Experiment 1 presented above were excluded for giving incorrect responses in around 50% of trials). If one cannot rely on chance-level performance as an exclusion criterion, it is common to include “catch” trials during the experiment – trials that will unambiguously indicate whether the participant was paying attention (for example, in an experiment where participants need to give their intuitions about the multiplication of 3-digit numbers, one could use the multiplication of single digit numbers as a control; another example would be including trials which would say, for example, “Press M” when *M* is not one of two regular response keys in the experiment). One would then exclude participants who do not reach a certain level of performance in these catch trials regardless of what responses they give in the rest of the experiment.

Even if participants do respond correctly, we still need to make sure that they have followed the instructions precisely (for example, in experiments reported here they should have read that their task is to respond as quickly as possible). One way to make sure this happens is to exclude everyone who read the instructions for less than a certain amount of time that is considered by the researcher to be sufficient^11^ (e.g., one participant in Experiment 1 and two participants in Experiment 2 of the present paper were excluded for reading the instructions for less than 10 seconds). Another way is to ask the participants to respond to several quick questions about the instructions before they proceed further in the experiment.^12^ Finally, yet another common way to identify dishonest participants is to include a question asking how honest they were at the very end of the experiment, informing them that the response they give will not affect whether they receive payment.

### Ensuring participants do not get distracted

A common worry is that participants will be multi-tasking during the experiment when we in fact would like them to be focused only on the task at hand (for example, it seems to be common for Amazon Mechanical Turk workers to watch TV or listen to music while doing experiments, see Chandler et al., 2014; Necka et al., 2016). To mitigate this issue, one can strive to administer shorter experiments (for example, no longer than 20 minutes) in order to decrease the chance of participants getting bored and wanting a distraction. This has the consequence that one cannot include many trials and will, therefore, have fewer datapoints per participant which can potentially be compensated for by collecting data from more participants (for example, this is how I solved this issue for Experiment 2, above; but see Baker et al., 2019; Brysbaert, 2019; Brysbaert & Stevens, 2018 for a discussion of the trade-off between participants and number of experimental trials for statistical power). In addition, there are ways to ensure attention while the experiment is running. For example, I make my experiments auto-paced: trials start and end regardless of whether participants press any buttons (e.g., after 2000 ms of no response, the next trial starts), so the participants do not have an opportunity to divert their attention to something else. Similar to excluding participants if they read the instructions for too short a period, one could also exclude participants if they spend too long on the break between blocks: if someone takes a 10 minute break after 5 minutes of doing the task, they were likely distracted.

### Ensuring participant naivety

As mentioned, non-naivety is not a large problem for typical cognitive psychology research, and is, therefore, not likely to be a problem for numerical cognition research either (Zwaan et al., 2018). However, one could in principle restrict participation to participants who have completed fewer than a certain number of studies on the participant recruitment service that is used (for example, it is possible to do so on Prolific, and I did so in Experiment 2 above; note, however, that this does not exclude participants who may have completed many studies through another participant recruitment service).

### Transparent reporting

As is clear from all the possibilities laid out above, there are a multitude of criteria that one can use to exclude certain participants’ data from analyses. These researcher degrees of freedom (Simmons, Nelson, & Simonsohn, 2011) are arguably somewhat larger than in the case of a traditional lab-based data collection, so in the case of web-based experiments it is even more important to preregister the planned exclusion criteria in order to avoid making (conscious or unconscious) biased decisions about data exclusion (Nosek, Ebersole, DeHaven, & Mellor, 2018; Wagenmakers, Wetzels, Borsboom, van der Maas, & Kievit, 2012).

### Web-based data collection with children

Everything discussed in this paper so far applies largely to data collected from adult participants. A substantial amount of research in numerical cognition, however, investigates the development of numerical abilities in children, where web-based data collection methods can also be effective in reaching large sample sizes. Unfortunately, the process for collecting developmental data in web-based experiments does not yet seem substantially easier than traditional developmental studies. I will highlight two issues here (for experience with web-based data collection with children and further discussions see Irvine, 2018; Nation, 2018).

Unlike adults, children cannot find and start a study themselves and would not do so in exchange for payment. This means that either a parent or a teacher has to be recruited, start the study and ask the child to complete it. Thus, in this case one still needs to find partner schools that can help with data collection or parents willing to take part in the project. If cooperating teachers are found and helped to go through the process of starting the study and passing it on to the child, they can repeat it without the researcher’s presence later, making this data collection method especially useful for longitudinal studies and studies that can be given to children in multiple school years. Parents could potentially be recruited through social media, in which case the parent should be able to go through the starting-up process and then convinced to not interfere when the child is completing the task. Importantly, one could also administer a home numeracy environment questionnaire to the parent before or after the child completes the task.

There is also an issue with consent, which usually needs to be given by a legal guardian. What is and what is not allowed in this respect will need to be determined by the researchers’ local ethical committee. Obtaining consent may not be an issue if a parent starts the experiment and passes it on to the child. For studies administered at school, depending on the nature of a task (whether it falls within a normal set of tasks a child would do at school), level of anonymity of the collected data, and the ethical board stance, one could consider consent as having been given by the teacher when they started the experiment and passed it on to the child.

Despite these additional complications, a number of web-based studies with children are currently being undertaken (e.g., Irvine, 2018; Nation, 2018), including at least one on number cognition (Callaway, 2013).

## Conclusion

In this paper, I have outlined the potential advantages and issues with web-browser-based data collection in numerical cognition research. I have also provided pointers for solving practical issues for those starting out with web-based data collection. The successful replications presented here demonstrate that it is not only *possible* to conduct such experiments, but they also yield comparable data quality. Of course, not every type of a study can be conducted in web-browsers, but one would be wise to choose this method for studies that *can*, since it saves time and money as well as possibly providing better and larger participant samples. Finally, I have offered some tips for ensuring good data quality. While every study will be unique in the ways in which better data quality can be achieved, by making some adjustments to the ways in which we are trained to ensure data quality, it should be possible to come up with ways to check that participants pay attention, complete the study honestly, etc. for many of the cases.

One final point to address is how we should deal with cases where we will not observe an effect in a web-based study – can we trust it or perhaps it was due to certain timing inaccuracies in web-based data collection? This problem is the same as in case of observing a null result in a lab-based study. The difference is only that in a lab-based study we have presumably eliminated inaccuracies in timing of stimuli so we are more confident that such a null result is due to the behavior of participants themselves. How do we deal with a null result in a web-based study? In the same way as we would in a lab-based study. For example, one solution would be to design experiments in such a way as to be able to observe a known control effect along with our null-result as a way to make sure the set-up is able to detect an effect of a certain size; another solution would be to move away from null hypothesis significance testing framework towards Bayesian analysis methods that allow to quantify the amount of evidence in favor of the null hypothesis. In general, if a researcher is skeptical about the validity of the results obtained in a web-based study, because they only require a web-browser to run, the same experimental scripts can be used both in web-based data collection and in physical lab spaces. One could administer (a part of) the experiment to a smaller sample of participants in a physical lab to verify the obtained result from the web-based study. Overall, a combination of web-based and lab-based data collection methods (verifying the patterns obtained with one by collecting data with another method) would lead to higher confidence in presence of the effect and its generalizability to a larger population.

## Data Accessibility Statement

Raw data, data collection scripts and data analysis scripts for both experiments presented in this manuscript are publicly available: https://osf.io/dy8kf/, DOI 10.17605/OSF.IO/DY8KF.

## Footnotes

Approximately 9% of articles published in the first issue of *Journal of Cognition* and 9% of the talks presented at *ESCoP 2019* conference fall under this research area (based on my informal calculation).

Just 1–3.2% of Google Scholar hits for search terms “numerical cognition”, “numerical magnitude”, “number processing”, “approximate number system” published between 2017–2019 (as of July 2019) also mention Amazon Mechanical Turk or Prolific Academic (two most popular online participant recruitment services), whereas estimated 11–31% of articles in cognitive and experimental psychology journals in general did so already in 2017 (Stewart et al., 2017).

Note that, as this is a fast-growing industry at the moment, many new tools are being released and some existing tools are being discontinued, so I do not attempt to list them all here.

Keep in mind, however, that in this case participants are not going to be specifically setting aside time and ensuring they are in a quiet environment to take part in a scientific experiment; the experiment would rather be a quick entertainment for participants.

I also hope that these supplemental materials can serve as an example of how web-based data collection can foster transparency and reproducibility (as discussed above) in numerical cognition research.

Note that Henik and Tzelgov (1982) do not mention any data exclusion, but I did it in this experiment because this experiment was part of a larger project where we applied uniform data processing across all experiments. However, I ran the same analysis of data from Experiment 1 without excluding any reaction times, and the results are the same as the ones presented here.

I opted for depicting the results in two separate plots because a large overlap between the lines within the same plot would hinder visibility of the patterns.

The effect sizes that I report here are based on the reported F-statistic in Henik and Tzelgov (1982), Experiment 2. Note that their analysis also included data from a different task, where participants compared the physical (font-) size of the digits instead of numerosity, so the effect sizes are not based on exactly the same analysis. Note also that their experiment had only 10 participants, so effect sizes estimated in their study have large confidence intervals around them.

To my knowledge, these are the only two published studies reporting a subliminal priming task in a web-based set-up.

The effect sizes that I report here and hereafter are based on the reported F-statistic in van Opstal et al. Note that the analyses in van Opstal et al included a second task, a letter task, so they do not reflect the effect in exactly the same analysis configuration.

Note that by *excluding* I do not mean not paying the participant, but simply excluding them from the analyses. They have contributed their time, and we can never know how well they actually read the instructions, so it is unjustified to withhold payment. On the other hand, I do not pay participants who have only chance performance in straightforward tasks, as in the experiments detailed in the present paper: in those cases, it is clear that they have not even tried to perform the study as instructed.

Both the time spent reading the instructions and the correctness of responses to questions about instructions can be automatically checked by the experiment script while the participant is doing the task, and from there the researcher can decide to either stop the experiment or to give the participant a second chance: for example, by informing them that it is important that they read and understand the instructions and displaying the instructions again.
